# Improvements in well-being following naturalistic psychedelic use and underlying mechanisms of change in older adults: A prospective cohort study

**DOI:** 10.21203/rs.3.rs-3977169/v1

**Published:** 2024-03-08

**Authors:** Hannes Kettner, Leor Roseman, Adam Gazzaley, Robin Carhart-Harris, Lorenzo Pasquini

**Affiliations:** Imperial College London; University of Exeter; UCSF; University of California San Francisco; University of California San Francisco

**Keywords:** aging, psychedelics, mental health, well-being, cohort study, naturalistic

## Abstract

Affective symptoms such as anxiety, low mood, and loneliness are prevalent and highly debilitating symptoms among older adults (OA). Serotonergic psychedelics are novel experimental interventions for affective disorders, yet little is known regarding their effects in OA. Using a prospective cohort design, we identified 62 OA (age ≥ 60 years) and 62 matched younger adults (YA) who completed surveys two weeks before, and one day, two weeks, four weeks, and six months after a guided psychedelic group session in a retreat setting. Mixed linear regression analyses revealed significant well-being improvements in OA and YA, amplified in OA with a history of a psychiatric diagnosis. Compared to YA, acute subjective psychedelic effects were attenuated in OA and did not significantly predict well-being changes. However, a psychosocial measure of *Communitas* emerged as a predictor in OA, suggesting that the relational components in psychedelic group settings may hold particular value for OA.

## Introduction

Emotions are central to human functioning, guiding thought and action from the earliest to the latest days of life (Carstensen et al., 1999). Emotional experiences change over the adult life span, with older adults (OA) shifting their motivational goals towards optimizing emotional regulation and reporting positive emotions more often than their younger counterparts (Carstensen et al., 2003) However, affective symptoms, such as anxiety, mood instability, loneliness, and apathy, are common among OA and may herald incipient neuropsychiatric and neurological disorders, such as late-life depression and Alzheimer’s disease (Conwell et al., 2023; Donovan & Blazer, 2020; Donovan et al., 2017; Rosenberg et al., 2013; Steffens, 2012). In particular, loneliness, or the subjective feeling of being socially isolated, has been identified as a major modifiable risk factor for cognitive decline and worsening of mental well-being in OA (Donovan & Blazer, 2020; Donovan et al., 2017; Luo et al., 2012). Loneliness has been shown to spread among social networks, to predict low life satisfaction, depressive symptoms, cognitive impairments, and Alzheimer’s disease dementia (Hawkley & Cacioppo, 2010), highlighting the importance of nurturing healthy social connections in the elderly. Crucially, there is convergent evidence that conventional antidepressants – including selective serotonin reuptake inhibitors – are less effective in treating affective symptoms in OA patient populations (Banerjee et al., 2011; Rosenberg et al., 2010). On the contrary, their use has been associated with an increased incidence of adverse respiratory and gastrointestinal events, as well as emotional blunting when compared to placebo (Banerjee et al., 2011; Carhart-Harris et al., 2021; Rosenberg et al., 2010), highlighting the need for improved treatments for affective symptoms in OA.

Serotonergic psychedelics, such as psilocybin (contained in “magic mushrooms and truffles”), lysergic acid diethylamide (LSD), N, N-Dimethyltryptamine (DMT, the main ingredients of the Amazonian brew ayahuasca), and mescaline, have recently garnered increasing research interest, following several clinical trials suggesting the therapeutic potential for these substances in the treatment of affective symptoms across various neuropsychiatric disorders (Andersen et al., 2021). Acute psychedelic effects are induced though the strong affinity of these substances for the serotonin 2A receptor (Vollenweider & Smallridge, 2022; Vollenweider et al., 1998); they include an altered state of consciousness characterised by intensified affect, vivified imagination and imagery, multisensory changes in perception, distorted sense of time, perceived spiritual and mystical experiences, and facilitated psychological insight. These latter effects, especially, have been indicated as mediators of therapeutic responses to psychedelics (Haijen et al., 2018; Kangaslampi, 2020; Roseman et al., 2019; Roseman et al., 2018).

Importantly, a recent review found that among 1400 participants enrolled in 36 psychedelic trials since 1967, only 19 participants (1.4%) were 65 years or older (Bouchet et al., 2024). The safety and efficacy of psychedelic treatments in older populations thus remains largely unknown (Johnston et al., 2023), although several authors have argued for the potential of psychedelics to loosen cognitive habits in old age generally (Pollan, 2019) as well as more formally as treatments for Alzheimer’s disease (Forester et al., 2022; Sarangi & Akinkunmi, 2023; Vann Jones & O’Kelly, 2020; Winkelman et al., 2023), including mild cognitive impairment (Steinberg et al., 2023) and even healthy cognitive decline and age-related affective changes (Aday, Bloesch, et al., 2020; Beaussant & Nigam, 2023). Initial clinical trials are currently underway investigating the effects of psychedelics on affective symptoms in patients with Alzheimer’s disease (NCT04123314) and Parkinson’s disease (NCT04932434).

Outside of the highly-controlled environment of clinical trials, psychedelic substances are commonly consumed recreationally, and increasingly also for self-medicative purposes (Bornemann et al., 2021; Mason & Kuypers, 2018; Matzopoulos et al., 2022). This includes guided individual and group settings, sometimes referred to as “ceremonies”, facilitated both underground and, in some countries, legally, e.g. at psychedelic retreats (Rucker & Young, 2021), where emphasis is placed on curating social contexts that foster interpersonal trust, openness, and expression of vulnerability (Kettner et al., 2021). This is typically achieved through encouraging the structured sharing of personal and subjective experiences in group circles before and after psychedelic sessions and by exercising compassionate non-judgemental listening, reflecting some of the principles also employed in group psychotherapy (Callon et al., 2021; Gearin, 2015; Tramacchi, 2000). As such, previous work has shown that psychedelic group settings can enhance psychological well-being and social connectedness by generating a sense of togetherness and self-disclosure within and outside of the acute substance sessions (Kettner et al., 2021).

Prospective observational studies of group ceremonies and other naturalistic psychedelic use can therefore be used to monitor psychological outcomes among psychedelic users in a more ecologically valid fashion and in more diverse populations. For example, a recent study following this approach has shown improved well-being among young adult (YA) and adolescent psychedelic users, as well as age-dependent differences in salutogenic mechanisms (Izmi et al., in prep). Yet, little is known regarding the potential of psychedelic experiences for improving mental health among OA. To investigate this question, we leveraged self-report data from an observational prospective cohort study of participants attending guided psychedelic group sessions (reported on also in Kettner et al., 2021).

## Materials and Methods

### Study design and participants.

The present study employed a prospective cohort design utilizing an online convenience sample of individuals planning to attend an organised psychedelic retreat or group-based guided psychedelic ceremony session, on their own accord. Recruitment took place via two routes: firstly, through online advertisements on psychedelic-related social media channels (Facebook groups, Twitter), email newsletters, and online forums (e.g., Reddit), and secondly, through retreat facilitators who advertised the study to their prospective clients. Participants were able to review study information online, provided informed consent, and subsequently completed surveys through multiple e-mails sent before and after their planned experience: 1) Within two weeks prior to the session, assessing demographics and baseline scores of mental health related outcome variables; 2) 24 h after the session, assessing acute subjective effects; 3) one day after leaving the ceremony or retreat location, including variables related to the overall experience; 4) two weeks, four weeks, and six months after the experience, measuring changes in the outcome variables. Eligibility criteria included being ≥18 years old, a good comprehension of the English language, and intentions to attend a psychedelic ceremony (i.e., involving use of psilocybin/magic mushrooms/truffles, LSD, ayahuasca, DMT/5-MeO-DMT, mescaline, or iboga/ibogaine). The study was approved by the Imperial College London’s Research Ethics Committee (ICREC) and the Joint Research Compliance Office (JRCO). For a full overview of the study design, see Kettner (et al., 2021).

Participants were included if they had completed at least the baseline and the 24h post-session survey. OA were identified based on a reported age of ≥60 and a set of matched YA was selected using nearest neighbour matching via the *MatchIt* package implemented in R (https://cran.r-project.org/web/packages/MatchIt/vignettes/MatchIt.html), matched for gender, education, psychiatric history, previous psychedelic use, baseline well-being, and drug dose.

### Measures

#### Baseline predictors.

At baseline, age, gender, education, extent of prior experience with psychedelic substances, expectations regarding potential beneficial effects of the experience (0–100 visual analogue scale), and self-reported history of psychiatric diagnoses were assessed, as well as basic information regarding the planned experience, such as substance type and location.

#### Outcome measures.

The Warwick-Edinburgh Mental Wellbeing Scale (WEMWBS) (Tennant et al., 2007)wasassessed at baseline and at the three endpoints two weeks, four weeks, and six months following the session to measures changes in mental well-being.

#### Acute psychedelic effects.

One day after the psychedelic session, measures of acute psychedelic effects were assessed. These included: (1) the Ego-Dissolution Inventory (EDI) (Nour et al., 2016), a measure reflecting the loss of a subjective experience of the self, which is typically induced by psychedelics, that has participants rate 10 items on a 0–100 scale; (2) the Mystical Experience Questionnaire (MEQ) (Barrett et al., 2015), a 30-item 6-point Likert scale measuring facets of mystical-type and peak experiences; (3) the Challenging Experience Questionnaire (CEQ) (Barrett et al., 2016), a 26-item 6-point Likert scale assessing difficult responses to the drug, such as fear, paranoia, and physiological alterations; (4) the Emotional Breakthrough Inventory (EBI) (Roseman et al., 2019), a six-item scale assessing emotional release and resolution of past trauma; and (5) the Communitas Scale (COMS) a 8-item questionnaire assessing acute relational experiences of togetherness and collective joy during psychedelic group sessions (Kettner et al., 2021).

#### Post-acute mediators.

One day post-retreat (on the day after leaving the ceremony location), participants completed: (1) the Psychological Insight Scale (PIS), assessing the degree to which the psychedelic experience was perceived as psychologically insightful via six 0–100 visual analogue scale items and (2) a version of the communitas scale (COMS_PR_), modified to assess relational experiences during the overall retreat, as opposed to the substance session only.

### Statistical analyses

Three mixed linear effects models, each including a random intercept, were used to assess changes in WEMWBS scores from baseline to two weeks, four weeks, and six months after the psychedelic session: a first model in OA, including only time point as a fixed effect, a second model in OA to which baseline demographic characteristics and their interaction with time points were added, and a third model comparing longitudinal changes in WEMWBS across OA and matched YA by including the interaction of time points and age group in the fixed portion of the model. Two-tailed paired t-tests were used to further assess significant WEMWEBS changes from baseline to each of the later time points in the whole OA sample and in a subsample of OA with a psychiatric diagnosis.

Next, we used MANCOVA and ANOVA models to compare the intensity of subjective acute psychedelic effects across the OA and YA groups. Additionally, a fourth mixed linear model with OA and matched YA was used to assess a three-way interaction between subjective acute psychedelic effects, age group, and changes in WEMWBS, aiming to expose age-related differences in salutogenic mechanisms. Pseudo-standardised regression coefficients (β) were calculated for this model to facilitate interpretability of the findings. Additional pairwise Pearson’s correlations between WEMWBS change scores from baseline to the four-week endpoint and subjective acute and post-acute psychedelic effects scores were calculated separately for OA and YA to illustrate the three-way interaction results in a simplified manner. A significance level of *p*<.05 was applied to all statistical tests. Where applicable in case of multiple comparisons, both uncorrected and Bonferroni-corrected levels of significance are reported.

## Results

### Demographic information

A total of 882 participants that signed up to the study, out of whom 819 provided baseline information. Among 106 participants that reported ≥ 60 years of age, 62 had completed the baseline and also the 24h post-session survey, yielding the final OA sample analysed in our study. Among these 62 OA, 53 completed the post-retreat questionnaire, evaluating experiences across the entire retreat period; 44 completed the two-week, 61 completed the four-week, and 23 completed the six-months endpoints. From a total of 430 adults with age < 60 years who completed at least the baseline and the 24h post-session survey, a set of 62 matched YA were selected using nearest neighbour matching (Table 1). For an overview of demographic information in the full YA sample, see **Supplementary Table S1**.

The mean age in the identified 62 OA was 65.1 years (SD = 4.02; range = 60–75) and exactly half (31/62, 50.0%) were male. A majority (43/62, 68.5%) of OA had a Master’s degree or higher, no history of diagnosed mental illness (46/62, 74.2%), and no prior experiences with psychedelics (35/62, 56.5%). Among OA who indicated psychiatric diagnoses, the most common were major depressive disorder (10/16) and anxiety disorder (9/16); alcohol dependence and ADHD were indicated by two individuals, respectively; personality, bipolar, and eating disorders by one person each. 59 OA attended psilocybin mushroom or truffle sessions (57 of which took place at retreat centres in the Netherlands or Jamaica), while three individuals indicated ayahuasca as the used psychedelic. Further, a sample of 62 YA (mean age in years (SD) = 46.5(10); range = 24–59) was selected via nearest neighbour matching for comparison purposes.

### Post-psychedelic mental health improvements

**Figure 1:**
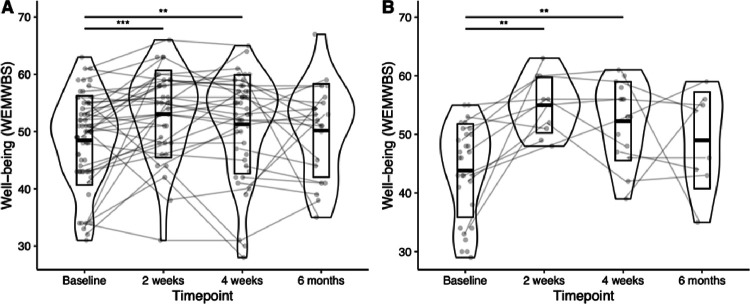
Mental well-being increases in OA following a psychedelic group session. Violinplots showing the distribution of WEMWBS scores in all OA **(A)** and OA with a lifetime psychiatric diagnosis only **(B)**at each time point, with lines reflecting individual trajectories. OA: older adults; WEMWBS: Warwick-Edinburgh Mental Wellbeing Scale; **: *p*<.01, ***: *p*<.001

A mixed effects linear regression model revealed WEMWBS increases in OA following the psychedelic session (Fig. 1A). An average increase of four points on the WEMWBS was found at the two-week endpoint (*B* = 4.09, 95% CI [1.87, 6.31], *p* < .001); this remained a three-point increase at the four-week endpoint (*B* = 3.05, 95% CI [1.04, 5.06], *p* = .004), indicating meaningful improvements in well-being. At six months post dosing, well-being scores were still nominally elevated by 1.7 units, which did not, however, reach significance (*B* = 1.72, 95% CI [−1.00, 4.44], *p* = .22). Paired *t*-tests comparing endpoint to baseline scores confirmed this pattern, with significant well-being increases at two weeks (*p* = .006, Cohen’s d = 0.48, *t*(34)= −2.87) and four weeks (p = .004, Cohen’s d = 0.44 *t*(45)= −3.01) but not at six months (*p* = .63, Cohen’s d = 0.10, *t*(20)=−0.48).

Having established that well-being improves in OA following psychedelic group sessions, we next investigated whether any individual demographic characteristics predicted post-psychedelic changes in mental well-being. We conducted a mixed effects linear regression model including age, gender, education, extent of prior experience with psychedelic substances, expectations on the beneficial effects of psychedelics, and history of mental illness as predictors. This model revealed only an interaction of history of mental illness with time (*B* = 6.62, 95% CI [1.53, 11.71], *p* = .019 at two weeks; *B* = 5.69, 95% CI [1.14, 9.94], *p* = .016 at four weeks), indicating that well-being increased more drastically in OA reporting a lifetime psychiatric diagnosis (Table 2). Paired t-tests within the subsample of OA with a psychiatric diagnosis (Fig. 1B) revealed significantly increased WEMWBS scores at two weeks (*p* = .007, Cohen’s d = 1.02, *t*(10)=−3.42), with an average increase of 9.4 points at four weeks (*p* = .004, Cohen’s d = 0.89 t(14)=−3.44) but not at six months (*p* = .48, Cohen’s d = 0.10, *t*(7)=−0.74).

We lastly aimed to assess whether changes in OA were comparable to those observed in YA. We conducted a mixed effects linear regression model with the OA and matched YA samples, which revealed no significant interactions between age group and time, indicating that well-being improvements in OA and YA were statistically indistinguishable in this sample (*B*=−2.36, 95% CI [−5.48, 0.76], *p* = 0.14 and *B*=−2.55, 95% CI [−5.53, 0.42], *p* = .097, for two weeks and four weeks post-dosing, respectively; **Supplementary Figure S1**).

### Comparing subjective psychedelic effects in OA and YA

**Figure 2:**
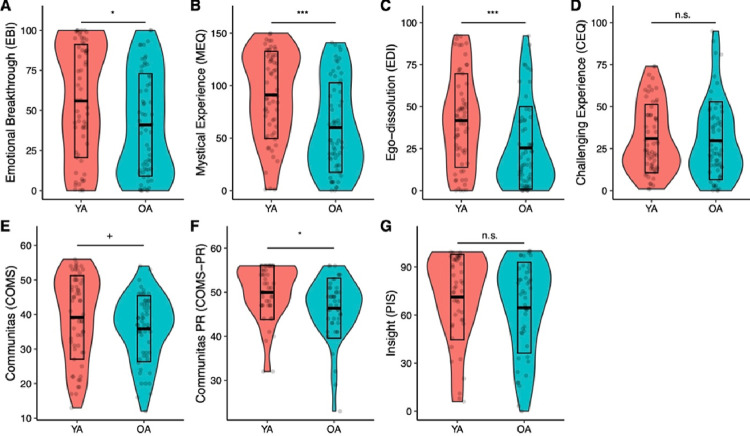
Boxplots showing intensity ratings of subjective psychedelic effects. Acute and post-acute effects were found to be attenuated in OA (> 60 years) compared to YA. OA: Older adults; PR: post-retreat; YA: Younger adults. +:*p*<.01, *:*p*<.05; **: *p*<.01; ***: *p*<.001

A MANOVA comparing ratings of acute subjective effects (EBI, MEQ, EDI, CEQ, COMS) between OA and YA revealed significant differences between the groups (Pillais’ Trace = .14, *F*(1,111) = 3.72, *p* = .004). Follow-up ANOVAs were then conducted (Fig. 2), revealing significantly lower intensity scores for OA on all tests included subjective effects measures, except for the CEQ (*F*(1,111) = 0.61, *d* = 0.06, p = .43) and only at trend level for the COMS (*F(1,111)* = 2.91, *d* = 0.30, p = .09), suggesting that OA experienced overall less intense subjective psychedelic effects compared to YA. The differences in mean scores between OA and YA were greatest for the EDI (48.6%, M = 25.4 vs 41.7, *F(1,111)* = 13.75, d = 0.62, *p* < .001) and the MEQ (39.1%, M = 60.1 vs M = 89.3, *F(1,111)* = 17.65, *d* = 0.74, *p* < .001), followed by the EBI (31.0%, M = 40.9 vs M = 55.9, *F(1,111)* = 5.44, *d* = 0.45, *p* = .02). Differences on the MEQ and EDI were significant also after Bonferroni-correction.

In a second MANOVA, post-acute effects, including insights (PIS) and Communitas experienced across the retreat as a whole (COMS-PR) were also found to be different by age group (Pillais’ Trace = .07, *F*(1,86) = 3.11, *p* = .05). Follow-up ANOVAs revealed this difference to be based on COMS-PR scores, which were significantly lower in OA (*F*(1,86) = 6.30, *d* = 0.55, *p* = .01), while PIS scores were not significantly different (*F*(1,86) = 0.27, *d* = 0.24, *p* = .60).

### Differential mechanisms predicting well-being changes in OA and YA

We subsequently explored whether age group-dependent acute and post-acute subjective psychedelic effects predicted long-term well-being outcomes via a mixed effects linear regression model including three-way interaction terms between age group, time, and each acute predictor variable. To prevent multicollinearity issues, variance inflation factors (VIF) for each included predictor variable were calculated, resulting in the removal of the MEQ from the model (VIF = 3.7) based on the generally accepted VIF cut-off of 2.5 (Johnston et al., 2018). After excluding MEQ, the highest VIF was found for EBI but beneath the established cut-off (VIF = 2.1). The resulting model revealed a significant negative three-way interaction between EBI, older age group, and post-psychedelic end-points at two- (β=−0.78, 95% CI [−1.43, −0.13], *p* = .02) and four-weeks (β =−0.80, 95% CI [−1.46, −0.15], *p* = .02), indicating that emotional breakthrough experiences contributed less to improved well-being in OA compared to YA. Furthermore, a marginally significant positive three-way interaction was detected for post-retreat COMS scores, OA, and the four-week end-point, suggesting that relational experiences of sharing and togetherness across the retreat played a larger role for predicting improved well-being in OA when compared to YA (β = 2.04, 95% CI [−0.21, 4.28], *p* = .08).

**Figure 3:**
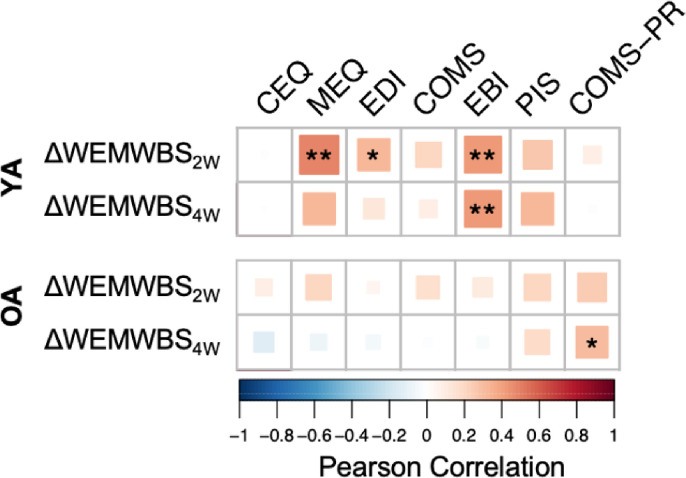
Correlation heatmap showing associations between subjective psychedelic effects and changes in well-being from baseline to two weeks (2W) and four weeks (4W) after a psychedelic group session. CEQ: Challenging Experience Questionnaire; COMS: Communitas Scale; EBI: Emotional Breakthrough Inventory; EDI: Ego-Dissolution Inventory; MEQ: Mystical Experience Questionnaire; OA: Older adults; PR: Post-retreat; YA: Younger adults. *:*p*<.05; **: *p*<.01

Correlation analyses between predictors and well-being change scores (Fig. 3) further illustrate these relationships: EBI was positively associated with well-being change scores at four-weeks in YA (r(35) = .43, *p* = .008), but not in OA (r(46)=−.04, *p* = .79), while post-retreat COMS-PR was positively associated with well-being change scores at four-weeks in OA (r(27) = .37, *p* = .03) but not in YA (r(34) = .01, *p* = .94). Interestingly, none of the other acute and post-acute variables that significantly correlated with well-being changes in YA were shown to significantly correlate with well-being changes in OA. Comprehensively, these findings suggest that the psychedelic experience fundamentally differs between OA and YA indicating a unique role for psychosocial experiences in the older group.

## Discussion

In this prospective study, we investigated the effects of naturalistic guided psychedelic group sessions on OA’s well-being by leveraging an opportunity sample of 62 participants aged 60 years or older attending self-initiated psychedelic ceremonies or retreats. Analyses revealed clinically meaningful improvements in well-being in OA at two and four weeks following a psychedelic group session, in line with prior naturalistic studies in YA (Kettner et al., 2021; Mason et al., 2019; Ruffell et al., 2021; Uthaug et al., 2021). Interestingly, this was the case despite lower acute subjective effects scores in the OA sample, indicating that differential salutogenic mechanisms may be at play in this age group. This exploratory hypothesis was partially confirmed through regression and correlational analyses showing a primacy of relational mechanisms, as opposed to classic intrasubjective psychedelic effects in OA attending psychedelic group sessions.

Among baseline and demographic variables predicting well-being increases in OA, only the presence of a psychiatric diagnosis showed significant effects. This finding was stable also when controlling for expectation effects, a hypothesized confounder in psychedelic trials (Muthukumaraswamy et al., 2021) and is in line with the transdiagnostic utility of psychedelic treatments for a number of mental health disorders (Brennan & Belser, 2022; Kelly et al., 2021; Kočárová et al., 2021), including major depression, alcohol-use disorder, and anorexia nervosa. Indeed, resilience to expectancy is consistent with recent research that failed to support its influence in driving therapeutic response to psilocybin therapy for depression (Szigeti et al., 2024), implying a substantive direct therapeutic action. Outside regulated clinical trial settings, the structured, user-reviewed services offered by retreat centres might have particular appeal to OA when compared to individual use (e.g., at-home). OA may have less access to or tend to avoid the acquisition of scheduled substances over the black market, may have greater psychological needs for safety, structure and social contact (Albert & Duffy, 2012), and the economical means to afford the often high financial cost of psychedelic retreats or ceremonies.

Crucially, clinically relevant improvements in mental health in OA were indistinguishable from those found in a sample of YA, matched to account for several demographic factors including higher OA well-being at baseline, a common finding in the literature (Carstensen et al., 2003). For example, elevated baseline well-being levels in OA are in accordance with representative population level studies showing that in wealthy English-speaking countries, happiness and hedonic experiences are lowest around ages 45–54 and tend to increase with age, following an inverted U-shape (Steptoe et al., 2015, Blanchflower, 2021). The observed return of well-being levels to baseline at the six-months follow-up time point in OA is in contrast with prior studies showing long-term mental health improvements following psychedelic-assisted psychotherapy (reviewed in Aday, Mitzkovitz, et al., 2020). Two prior naturalistic studies in YA have also found sustained two-year increases in protective psychological traits such as resilience and mindfulness (Mans et al., 2021), or nature relatedness (Kettner et al., 2019), although, similar to the present study, affective measures of well-being have thus far been shown to remain increased only at nominal, non-significant levels (Mans et al., 2021). The conditions under which psychedelic-induced salutogenesis remains stable therefore remains a critical unanswered question, considering that in clinical studies, improvements appear to remain significant for months to years following treatment (Agin-Liebes et al., 2020; Carhart-Harris et al., 2018; Gukasyan & Nayak, 2021; Johnson et al., 2017).

Our study revealed differential acute psychedelic effects and salutogenic mechanisms in OA when compared to YA. This is of clinical importance, since current models of psychedelic function propose that the acute psychedelic effects are key mediators of mental health improvements (Griffiths et al., 2008; Griffiths et al., 2006; Ko et al., 2022; Majić et al., 2015; Roseman et al., 2018). In contrast to prior controlled research reporting challenging experiences to be negatively correlated with age (Ko et al., 2023; Kopra et al., 2022; Studerus et al., 2012), the OA group in the present sample showed lower acute effects scores on all metrics except for challenging experiences. One potential reason for this apparent discrepancy may be the overall younger age (means ranging from 27–36) and lack of participants aged 60 or above in the abovementioned controlled studies. It is thus possible that the intensity of challenging experiences under psychedelics peaks among the younger distribution of YA and remains stable after a certain age, pointing to population diversity as a key strength of naturalistic studies such as this one.

Nonetheless, the absence of any significant correlations between acute psychedelic effects and long-term changes in OA contradicts previous work showing that the quality of the acute experience constitutes a key predictor of psychedelic-induced changes in well-being (Haijen et al., 2018; Kangaslampi, 2020, 2023; Kettner et al., 2021; Peill et al., 2022; Roseman et al., 2019; Roseman et al., 2018). In contrast, only the experience of Communitas rated in reference to the entire retreat – not just the psychedelic session – was associated with well-being changes in OA. This finding suggests that OA might benefit from psychedelics for different reasons than YA, greater relevance being given to the experience of togetherness and social bonds that can occur in group settings than to the individual, intrapersonal experience. The witnessing of other participants’ vulnerability and the resulting emotional intimacy generated through sharing rounds before and after dosing sessions might be particularly impactful to OA, for whom social contact, especially with nonfamily members, is known to decrease (Sander et al., 2017). Indeed, from the present data it is unclear to what extent the consumption of the psychedelic substance itself would have even been necessary for OA to experience the detected psychological benefits. Future research should thus further explore the details of psychotherapeutic and group activities taking place at psychedelic retreats, and their psychological benefits for participants, as well as the validity of instruments assessing the overall experience in OA. Conceivably, the psychedelic session itself could be seen as a non-essential part, primarily providing the context for an intimate and intergenerational group-based intervention with the potential to tackle the negative emotional and cognitive health consequences of social isolation in the elderly (Krzeczkowska et al., 2021; Murayama et al., 2015).

Further, the present findings of reduced acute psychedelic effects and increased importance of social connections may relate to the consolidation of “emotional landscapes” in OA (Carstensen et al., 2003). Our findings are in line with Carstensen’s (et al., 1999) Socioemotional Selectivity Theory posing that OA optimize emotional experiences to prioritize meaningful social connections and foster positive experiences and emotional satisfaction. Intriguingly, reduced acute psychedelic effects in OA may mechanistically also relate to age-dependent reductions in cortical serotonin receptor density, which is most pronounced for the 2A receptor (Karrer et al., 2019), the primary target of psychedelics (Madsen et al., 2019; Vollenweider et al., 1998).

Several limitations need to be considered when interpreting our findings. Most importantly, the context of psychedelic use in the present sample was limited to ceremony and retreat settings, raising the question whether well-being improvements, attenuated acute psychedelic effects, and greater importance of psychosocial mechanisms detected in the current sample would also occur in other naturalistic or controlled psychotherapeutic settings. Replications in larger and more representative samples will therefore be crucial to further explore the effects of psychedelic on the elderly outside psychedelic ceremony and retreat settings, and in samples less biased towards white, highly educated participants. Controlled laboratory studies administering psychedelics to OA will potentially be able to clarify the role of acute psychedelic effects in environments that do not provide the psychosocial group benefits present at psychedelic retreats. Additional limitations include the inaccurate qualitative assessment of psychedelic dose, as well as possible co-use of other substances - common in naturalistic samples (Kuc et al., 2021; Zeifman et al., 2023) - which was not controlled for in the present study. Furthermore, the potential of systematic biases through attrition effects constitutes another limitation related to the remote nature of this survey study, although prior research has shown that attrition in prospective psychedelic surveys follows similar patterns as in other fields (Hübner et al., 2021).

## Conclusions and future directions

Echoing previous observational studies in YA and clinical trials, our findings suggest that psychedelic group sessions may induce rapid and sustained benefits on OA’s well-being, especially in those with a history of a psychiatric illness. While further replication and controlled studies are necessary, the present findings further suggest that OA might experience an overall attenuated intensity of acute psychedelic effects, and that acute effects might be less predictive of prospective changes in mental well-being. Instead, the greater importance of psychosocial experiences indicate that the group setting may be particularly valuable for OA. Our findings represent a first inquiry into the factors that contribute to the enduring nature of salutogenic psychedelic effects in OA and call for longer-term observational studies and clinical trials in OA.

## Figures and Tables

**Table 1:** Demographic characteristics of the study samples. OA: older adults; SD: Standard Deviation; WEMWBS: Warwick-Edinburgh Mental Wellbeing Scale; YA: younger adults

	OA (N = 62)	YA matched (N = 62)
**Age in years**		
Mean (SD)	65.1 (4.02)	46.5 (10.0)
Median [Min, Max]	64.0 [60.0, 75.0]	49.0 [24.0, 59.0]
**Gender**		
Male	31 (50.0%)	31 (50.0%)
Female	31 (50.0%)	31 (50.0%)
Other	-	-
**Education/degrees**		
None	1 (1.6%)	0 (0%)
High school	3 (4.8%)	2 (3.2%)
Technical degree	4 (6.5%)	5 (8.1%)
College diploma	11 (17.7%)	18 (29.0%)
Master	19 (30.6%)	30 (48.4%)
PhD/MD/Law degree	24 (38.7%)	7 (11.3%)
**Psychiatric diagnoses**		
Yes	16 (25.8%)	15 (24.2%)
No	46 (74.2%)	47 (75.8%)
**Psychedelic use #**		
Never	35 (56.5%)	28 (45.2%)
1–5 times	15 (24.2%)	10 (16.1%)
> 5 times	12 (29.3%)	24 (38.7%)
**WEMWBS baseline**		
Mean (SD)	48.5 (7.79)	48.8 (9.79)
Median [Min, Max]	50.0 [31.0, 63.0]	50.5 [20.0, 70.0]
**Psychedelic dose**		
Mean (SD)	2.10 (0.646)	2.13 (0.586)
Median [Min, Max]	2.00 [1.00, 3.00]	2.00 [1.00, 3.00]

**Table 2:** Main effects and two-way interactions of baseline and demographic variables on wellbeing across time in OA. f: female; SE: Standard Error.

Term	*B*	SE	t-value	*p*
Intercept	19.28	17.99	1.071	.28
2-week endpoint	9.77	18.42	0.530	.60
4-week endpoint	15.74	16.39	0.960	.34
Age	0.39	0.26	1.502	.14
Gender (f)	−1.44	2.12	−0.681	.50
Psychedelic use	0.61	0.67	0.914	.36
Psychiatric history	−3.00	2.45	1.207	.23
Expectations	.01	.06	0.258	.80
Age:2-week endpoint	0.00	0.27	−0.016	.99
Age:4-week endpoint	−0.17	0.23	−0.734	.47
Gender (f):2-week endpoint	−0.82	2.21	−0.373	.71
Gender (f):4-week endpoint	2.46	1.96	1.259	.21
Psychedelic use:2-week endpoint	−0.70	0.65	−1.083	.28
Psychedelic use:4-week endpoint	0.06	0.57	0.097	.92
Psychiatric history:2-week endpoint	−6.62	2.77	−2.388	**.02**
Psychiatric history:4-week endpoint	−5.69	2.32	−2.457	**.02**
Expectations:2-week endpoint	0.02	0.06	0.403	.69
Expectations:4-week endpoint	0.02	0.05	0.4000	.69

**Table 3:** Three-way interactions showing differential salutogenic mechanisms for OA and YA. For a full tabulation of main and two-way interaction effects, see Supplementary Table S2. CEQ: Challenging Experience Questionnaire; COMS: Communitas Scale; EBI: Emotional Breakthrough Inventory; EDI: Ego-Dissolution Inventory; PR: Post-retreat; PIS: Psychological Insight Scale; SE: Standard Error.

Term	β	SE	t-value	p
EDI:Age 60+:2-week endpoint	0.03	0.12	0.25	.80
EDI:Age 60+:4-week endpoint	0.02	0.11	0.217	.83
EBI:Age 60+:2-week endpoint	−0.22	0.10	−2.354	**.02**
EBI:Age 60+:4-week endpoint	−0.21	0.09	−2.416	**.02**
CEQ:Age 60+:2-week endpoint	0.08	0.11	0.788	.43
CEQ:Age 60+:4-week endpoint	0.09	0.10	0.855	.39
COMS:Age 60+:2-week endpoint	0.18	0.25	0.727	.47
COMS:Age 60+:4-week endpoint	−0.06	0.24	−0.24	.81
COMS_PR_:Age 60+:2-week endpoint	−0.02	0.38	−0.04	.97
COMS_PR_:Age 60+:4-week endpoint	0.68	0.38	1.793	.08
PIS:Age 60+:2-week endpoint	0.00	0.02	−0.16	.87
PIS:Age 60+:4-week endpoint	0.00	0.02	−0.234	.82

## Data Availability

Raw data can be shared from the corresponding author following reasonable request.
